# Effects of subclinical inflammation on C-reactive protein and haptoglobin levels as well as specific humoral immunity in dogs vaccinated against canine distemper and parvovirus

**DOI:** 10.1186/s12917-018-1383-6

**Published:** 2018-03-05

**Authors:** Przemysław Romiszewski, Krzysztof Kostro, Urszula Lisiecka

**Affiliations:** 1 Department of Epizootiology and Clinic of Infectious Diseases, Faculty of Veterinary Medicine, University of Life Sciences in Lublin, 20-612 Lublin, Poland; 2 Animal Medical Center, 5255 York Rd, P.O. Box. 324, Holicong, PA 18928 USA

**Keywords:** Dogs, Vaccine, Specific humoral immunity, Subclinical inflammation, C-reactive protein, Haptoglobin

## Abstract

**Background:**

The aim of the present study was to assess the effects of subclinical inflammation on specific humoral immunity in dogs vaccinated with Nobivac® DHP based on serum levels of CRP and Hp. Dogs from the group I were administered Nobivac® DHP, the vaccine against distemper, infectious hepatitis and parvovirus whereas group II animals received subcutaneous turpentine oil to induce subclinical inflammation, followed by Nobivac® DHP after 24 h. Animals in group III received only turpentine oil in the way and amount identical to that as in group II.

**Results:**

Nobivac DHP relatively poorly induced the immune inflammatory response showing good immunogenic properties, which was evidenced by only a double increase in mean CRP and Hp levels associated with antigenic stimulation in group I. In group II, serum neutralization (SN) and haemagglutination inhibition (HI) results were quite closely correlated with serum levels of CPR and Hp.

**Conclusions:**

Our findings suggest that the efficacy of vaccinations in dogs can be significantly affected by subclinical inflammations, which is indicated by a correlation between serum CRP and Hp levels versus antibody titres for canine distemper and parvovirus in both experimental groups of dogs (group I and II). The correlation of mean CRP and Hp values in dogs with subclinical inflammation and after vaccination with the kinetics of increasing antibody titres against distemper and parvovirus in group II dogs reflects the severity of inflammatory response and the extent of specific humoral immunity. Routine determinations of serum CRP and Hp levels as the indices of inflammation severity can be the essential biochemical markers for assessment of dogs’ health in the period preceding specific immunoprophylaxis and efficacy of the vaccine.

## Background

The extent and nature of post-vaccination immunity are strictly dependent on the vaccine composition and route of administration, possible suppressive effects of vaccine components and health of vaccinated animals [1, 16, 17, 19–21, 23, 24]. The efficacy of vaccinations is markedly affected by autoimmune diseases, acquired and congenital immunodeficiency, impaired hormonal profiles of immunized animals, particularly thyroid dysfunction, neoplastic diseases, infections with immunosuppressive viruses and environmental factors such as inappropriate living conditions, stress, mineral-vitamin deficiencies, food additives and immunosuppressive drugs [4, 5, 12, 15, 18, 22]. The above conditions result in increased susceptibility to infections, especially with opportunistic microorganisms, and low efficacy of properly administered specific immunoprophylaxis.

To date, the data concerning the relation between vaccination outcomes and negative effects of subclinical inflammations have not been numerous. The aim of the present study was to assess the influence of subclinical inflammation on specific humoral responses in dogs vaccinated against canine distemper and parvovirus based on serum levels of C-reactive protein (CRP) and haptoglobin (Hp).

## Methods

### Animals

The study encompassed 21 mongrels from a private kennel and informed consent was obtained from the owner. Dogs were chosen for the study based on clinical examinations, hematological results and parasitic status clearance. Nine dogs were excluded from the study because they failed to meet inclusion criteria.

The dogs, aged above 12 weeks, of both genders and comparable body weight, were divided into two experimental groups (group I and II) and control group (group III), 7 individuals each. Group I dogs were administered Nobivac® DHP, the vaccine against distemper, infectious hepatitis and parvovirus whereas group II animals received subcutaneous turpentine oil (1 ml/10 kg b.w.) [9] to induce subclinical inflammation, followed by Nobivac® DHP after 24 h. Animals in group III received only turpentine oil in the way and amount identical to that as in group II.

Serum levels of CRP and HP were determined in animals of group I, II and III; in group I and II, increases in specific antibodies against distemper and parvovirus were also analysed. The levels of CRP and Hp were measured on day 0, 1, 2, 3, 7, 14, 21 and 28 whereas the levels of specific antibodies against distemper and parvovirus on day 0, 7, 14, 21 and 28 after induction of inflammation and/or vaccination.

### Vaccine

The polyvalent vaccine Nobivac® DHP (Intervet) was used containing attenuated strains of canine distemper virus (CDV) (strain Onderstepoort), canine adenovirus type 2 (CAV2) and canine parvovirus (CPV) (strain Manhattan LPV 3). The vaccine was administered according to the manufacturer’s recommendations.

### Acute phase proteins (APPs)

Serum levels of CRP were determined using ELISA (Canine CRP, Tridelta Dev.t Ltd., Ireland) and of Hp using non-species specific, colorimetric assay for the determination of haptoglobin (Tridelta Dev.t Ltd.*,*). The concentration of CRP was expressed in μg/ml and of Hp in mg/ml.

### Specific humoral response

The titres of specific antibodies in serum were determined by the β-seroneutralisation (SN) test on Nunc microplates (Denmark) using the increasing twofold dilutions of sera previously thermally inactivated at 56 °C/30 min. Each serum dilution was mixed with a constant dose of parvovirus (100 CCID_50_), stored at 4 °C for 48 h and at 37 °C for 1 h. The mixture was applied to inoculate the continuous line cells CCC clone 81, using 8 plate wells per each serum dilution. The SN titres were determined based on the inverse of the highest serum dilution resulting in cytopathic effect inhibition for 50% of inoculated cell cultures.

The levels of antibodies inhibiting haemagglutination directed against parvovirus were determined with the β haemagglutination inhibition (HI) method using increasing twofold dilutions of sera mixed with 4 units of HA virus. Non-specific haemagglutination inhibitors (NSIs) and natural haemagglutinins were removed by thermal inactivation of sera (56 °C for 30 min.), absorption with 25% kaolin and saturation with pig erythrocytes.

Results were statistically analysed by calculating a mean and standard deviation; significance of intergroup differences was verified using the Duncan’s test and SAS software.

## Results

### CRP and Hp levels

**Fig. 1:**
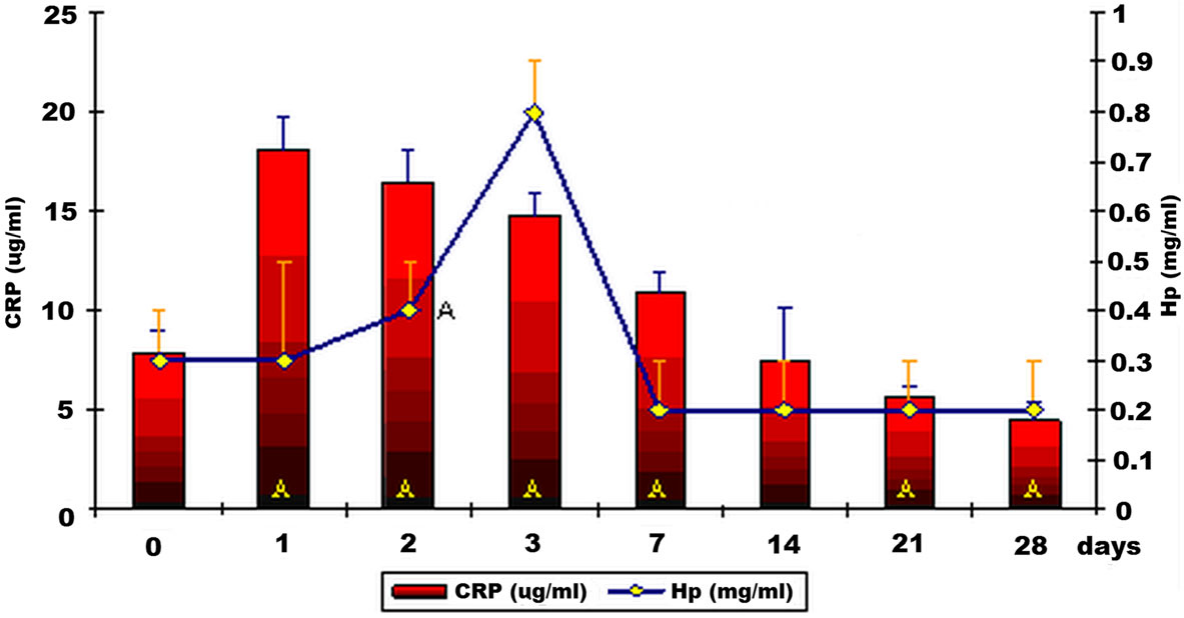
Serum levels of CRP and Hp in dogs vaccinated with Nobivac® DHP (α+/− SD) (group I). A – statistically significant differences compared to baseline values (*P* < 0.01)

In group I, mean serum levels of CRP and Hp on day 0 were low (Fig. 1); a significant increase in CRP (*P* < 0.01) was observed between post-vaccination day 1 and 7 as compared to baseline values, with the highest mean value (18.1 ± 1.7) found on post-vaccination day 1. Mean CRP values at the final two measurement points were significantly lower (*P* < 0.01) than baseline values. The statistically significant (*P* < 0.01) and highest increase in serum Hp levels was noted on post-vaccination day 3. Mean Hp levels at the final four measurement points were comparable to baseline values (Fig. 1).

**Fig. 2:**
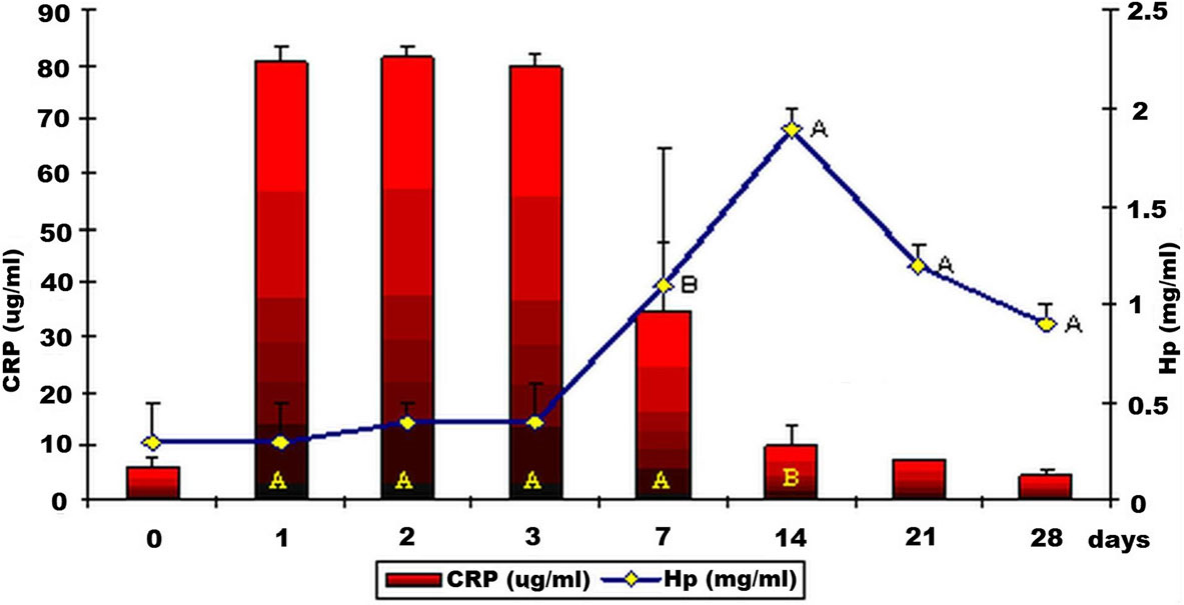
Serum levels of CRP and Hp in dogs with subclinical inflammation and vaccinated with Nobivac® DPH (α+/− SD) (group II). A – statistically significant differences compared to baseline values (*P* < 0.01). B – statistically significant differences compared to baseline values (*P* < 0.05)

In group II, a statistically significant (*P* < 0.01) CRP increase was observed between day 1 and 14 after experimentally induced inflammation and vaccination (Fig. 2). The highest mean values of CRP were noted on day 1, 2 and 3, i.e. 80.4 ± 3.2, 81.4 ± 1.9 and 79.7 ± 2.2 μg/ml, respectively. At the final two measurement points, mean concentrations of CRP were comparable to baseline values. Mean values of Hp in this group were comparable to baseline ones for 3 consecutive days to increase significantly (*P* < 0.05) on day 7 (Fig. 2). On day 14, Hp reached the maximum value (1.9 ± 0.1 mg/ml) and subsequently gradually decreased, yet remained higher throughout the experiment compared to day 0.

**Fig. 3:**
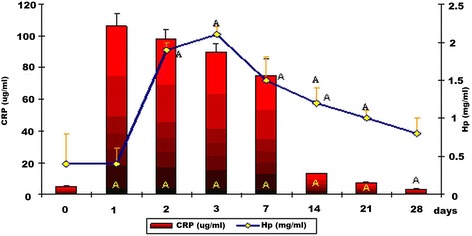
Serum levels of CRP and Hp in dogs with subclinical inflammation (group III) (α+/− SD). A – statistically significant differences compared to baseline values (*P* < 0.01)

In the group with experimentally induced inflammation (group III), the mean CRP value on day 0 was found to be low – 5.1 ± 0.4 μg/ml (Fig. 3). The maximum and statistically significant CRP level (*P* < 0.01) compared to the baseline one was observed on post-induction day 1 (106.3 ± 7.8 μg/ml). Subsequently, its mean concentration gradually decreased and on the last observation day it was lower than the baseline value. Furthermore, a significant increase in Hp (*P* < 0.01) in this group was noticed on post-induction day 2 reaching its highest mean value at the next measurement point (2.1 ± 0.1 mg/ml). Subsequently, mean Hp values decreased albeit remained significantly higher (*P* < 0.01) than baseline values (Fig. 3) at each measurement point.

### SN and HA titres

**Fig. 4:**
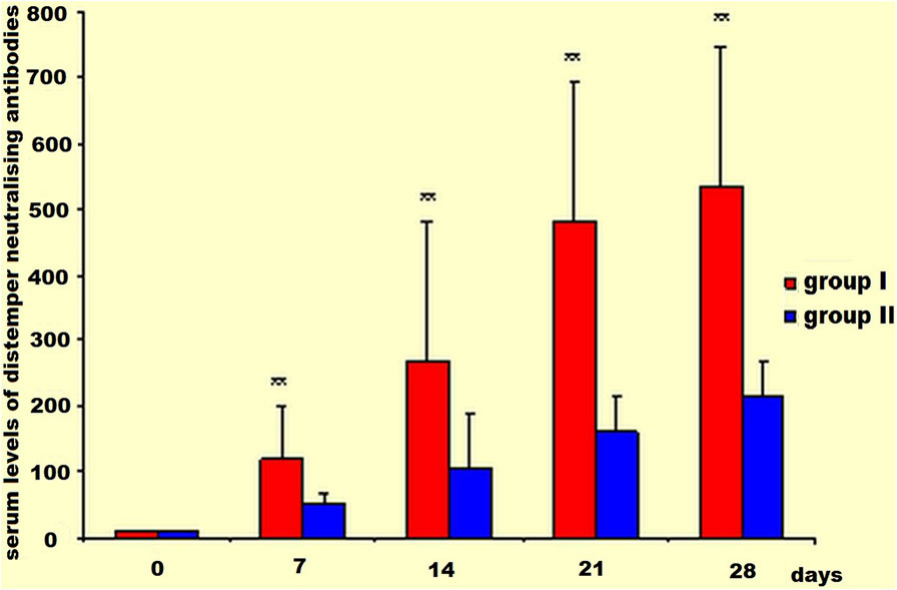
Serum levels of distemper neutralising antibodies in group I and II (α ± SD). Intergroup comparison at the same time intervals. ** - *P* < 0.01

**Fig. 5:**
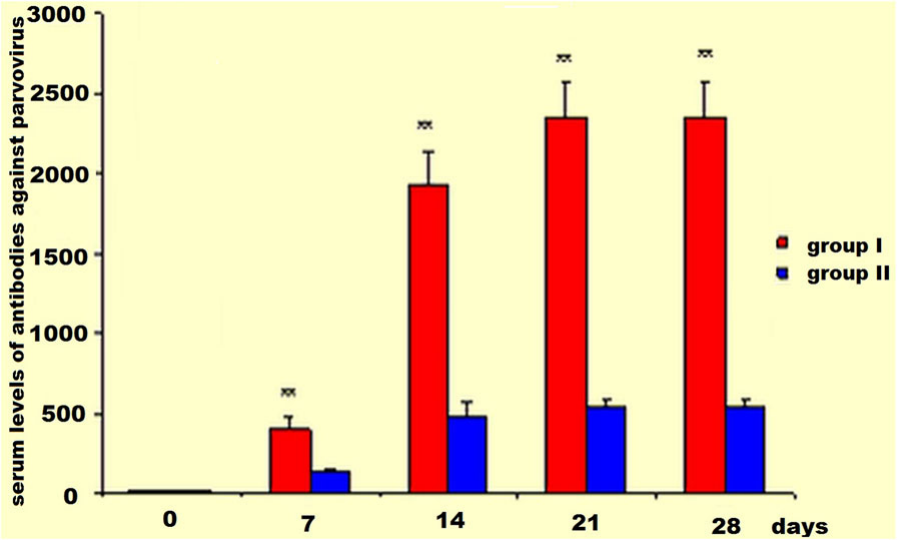
Serum levels of parvovirus haemagglutination inhibiting antibodies in group I and II (α ± SD). Intergroup comparison at the same time intervals. ** - *P* < 0.01

Positive titres of antibodies neutralising distemper and inhibiting haemagglutination of parvovirus were observed on post-Nobivac® DHP day 7 in all dogs of both experimental groups; at the next measurement points they further gradually increased (Figs. 4 and 5). However, there were significant differences in positive SN and HI results between group I and II at all measurement points; their magnitude was correlated with serum levels of CRP and Hp. During the entire observation period, the highest SN and HI titres in group I and II were found on post-vaccination day 28. The kinetics of increases in titres of SN and HI antibodies in both experimental groups was similar yet their mean values were significantly lower (*P* < 0.01) in group II at each measurement point (Figs. 4 and 5).

## Discussion

Our study demonstrated that serum levels of CRP and Hp could reflect the degree of activation of the immune system in dogs with induced inflammation or with inflammation and after vaccination. It is however difficult to compare the obtained results as the literature lacks any data regarding the usefulness of determinations of acute phase proteins (APPs) as sensitive markers of the severity of asymptomatic inflammation in dogs. Low mean levels of CRP and Hp, within the physiological range, in dogs before Nobivac® DHP administration (day 0) indicate that dogs in the period preceding vaccinations were healthy and their immune system was not activated. A twofold increase in mean serum levels of these proteins during the first post-vaccination week evidences the existence of immune response. The correlation of CRP and Hp changes after vaccination with the kinetics of increasing titres of antibodies reflects the enhancement of inflammatory response associated with antigenic stimulation. However, Nobivac® DHP relatively poorly induced the inflammatory immune response, showing good immunogenic properties at the same time (Figs. 1, 4 and 5). The above finding was also confirmed by Yule et al. [24]. They observed that increased CRP and Hp levels after challenge in dogs earlier immunised against parvovirus were inversely proportional to the extent of specific immunity, which either evidences poor immunogenic properties of the vaccine or presence of persisting immunosuppression which developed prior to immunisation. Thus, serum levels of CRP and Hp, reflecting the immune system activation can be the main biochemical markers to assess the dogs’ health in the period preceding specific immunoprophylaxis and efficacy of the vaccine used.

The efficacy of vaccinations is likely to be markedly affected by subclinical inflammations, which is indicated by the correlation between serum CRP and Hp levels and specific humoral immunity against distemper and parvovirus. The maximum serum levels of CRP on day 1 and of Hp on day 3 after experimental induction of inflammation observed in controls demonstrate that CRP is the major whereas Hp a moderate APP in dogs [2, 3, 6, 7, 11, 13], which is additionally confirmed by the results in dogs with inflammation and after vaccination (group II, Fig. 2). In group II, significantly higher mean values of these proteins than baseline values were observed until day 14 (CRP) and 28 (Hp) whereas in group III until day 21 (CRP) and 28 (Hp), which resulted from long-term subclinical inflammation induced with administration of intramuscular turpentine oil. From Figs. 1 and 2, the conclusion could be drawn that in group I the mean values of CRP and Hp concentrations on the first and the third day of the experiment were 4 fold decreased comparing with group II values. In both groups, the kinetics of the immune response was not similar and the mean values of both Hp and CRP concentrations differed significantly between group I and II. In the final stage of the experiment the mean CRP values were congenial in both group I and II. However the mean values of Hp in group II were significantly higher than in group I till the end of the study which can be connected with interference of inflammatory process caused by turpentine oil with the effect of Nobivac® DHP vaccine.

The increases in CRP and Hp in group II and III are likely to be associated with their involvement in the regulation of inflammatory immune response and repair of inflammation-injured tissues [9, 13]. Normalisation of CRP concentrations in group II, which did not occur until the final two measurement points, reflects the gradual subsidence of inflammation. Thus, monitoring of serum CRP and Hp levels in dogs with subclinical inflammation and vaccinated against distemper and parvovirus, may be helpful to assess the efficacy of biopreparations.

The induction of specific immune response in dogs with experimentally induced inflammation and immunised against distemper and parvovirus was confirmed by SN and HI titres (Figs. 4 and 5). Immunosuppression develops during inflammation due to increased percentages of TCD8^+^ suppressor lymphocytes and direct inhibitory effects on Th1 cells producing cytokines supporting the specific humoral immunity. In the final study period, on the other hand, the SN and HI test results in group II were found to reach the highest values in the entire experiment. This can be explained by the subsidence of inflammation inhibiting the specific humoral immune response and Th2 dominance in this period, which is confirmed by the results in group I dogs which were administered the vaccine. During the subsidence of inflammation in group II dogs, there was a relatively close correlation between SN and HI results versus serum levels of CRP and Hp. The above proteins, exerting both pro- and anti-inflammatory effects, are involved in immune response regulation [13, 14]. Low levels of CRP enhance the proliferation of lymphocytes whereas its high concentrations inhibit lymphocyte blastogenesis [14]. Moreover, Hp, as an active ligand for CD11b/CD18 integrins, inhibits multinuclear cell migration and, once released due to their activation, inhibits the activity of granulocytes infiltrating the inflammatory focus. High levels of Hp inhibit lymphocyte blastogenesis, chemotaxis, phagocytosis and the process of intracellular killing in granulocytes. Furthermore, Hp modulates the synthesis of specific antibodies by regulating the proliferation of B lymphocytes [8, 10, 14].

## Conclusions

Determinations of serum CRP and Hp as markers of inflammation, introduced to routine diagnostic procedures, broaden the possibilities of canine health monitoring in the period preceding specific immunoprophylaxis.

Inflammation, even subclinical, can result in the development of an immunological risk group, in which vaccination fails to provide sufficient immunity and individual vaccination schemes are required to increase the vaccination efficacy.

Dogs with subclinical inflammation vaccinated with Nobivac® DHP tend to have weakened humoral immune responses, which can lead to shortened duration and extent of post-vaccination immunity.

## Abbreviations

CAV2: Canine adenovirus type 2
CDV: Canine distemper virus
CPV: Canine parvovirus
CRP: C-reactive protein
Hp: Haptoglobin
